# Trajectories of physical activity from mid to older age in women: 21 years of data from the Australian Longitudinal Study on Women’s Health

**DOI:** 10.1186/s12966-023-01540-z

**Published:** 2024-01-08

**Authors:** Yuta Nemoto, Wendy J. Brown, Gregore Iven Mielke

**Affiliations:** 1 School of Public Health, The University of Queensland, Brisbane, QLD 4006 Australia; 2 Department of Preventive Medicine and Public Health, Tokyo Medical University, Tokyo, 160-8402 Japan; 3 School of Health Innovation, Kanagawa University of Human Services, Kanagawa, 210-0821 Japan; 4 Faculty of Health Sciences and Medicine, Bond University, Gold Coast, 4229 Australia; 5 School of Human Movement and Nutrition Sciences, The University of Queensland, Brisbane, QLD 4006 Australia

**Keywords:** Mid-aged women, Physical activity, Trajectories, Temporal change, Determinants, Menopause, Number of births

## Abstract

**Background:**

Women’s physical activity varies across the adult lifespan. However, changes in physical activity among mid-aged women are not well understood. We analysed 21 years of data from women born in 1946–51 to identify: (1) trajectories of physical activity in the transition from mid- to old-age and (2) determinants of different physical activity trajectories.

**Methods:**

Data were from the 1946–51 cohort of the Australian Longitudinal Study on Women’s Health (*N* = 10,371). Surveys were mailed at three-year intervals from 1998 (age 47–52) to 2019 (age 68–73) to collect data on physical activity, sociodemographic factors (country of birth, area of residence, educational attainment, marital status, income management, paid work hours, living with children age < 18, providing care), health indicators (menopause status, BMI, physical and mental health, chronic conditions), and health behaviours (smoking, alcohol status). Group-based trajectory modelling was used to identify trajectories of physical activity. Multinomial logistic regression models were used to examine the determinants of physical activity trajectories.

**Results:**

Five trajectories were identified: Low-stable (13.3% of participants), Moderate-stable (50.4%), Moderate-increasing (22.2%), High-declining (7.7%), and High-stable (6.6%). Sociodemographic characteristics (area of residence, education, income management, living with children, and providing care) were determinants of physical activity trajectories, but the strongest factors were BMI, physical and mental health. Women who were overweight/obese and had poor physical and mental health were less likely to be in the High-stable group than in any other group. Changes in these variables (increasing BMI, and declining physical and mental health) and in marital status (getting married) were positively associated with being in trajectories other than the High-stable group.

**Conclusions:**

Although most women maintained physical activity at or above current guidelines, very low physical activity levels in the Low-stable group, and declining levels in the High-declining group are concerning. The data suggest that physical activity promotion strategies could be targeted to these groups, which are characterised by socioeconomic disadvantage, high (and increasing) BMI, and poor (and worsening) physical and mental health. Removing barriers to physical activity in these women, and increasing opportunities for activity, may reduce chronic disease risk in older age.

**Supplementary Information:**

The online version contains supplementary material available at 10.1186/s12966-023-01540-z.

## Introduction

Physical activity (PA) is a well-known protective factor for chronic diseases and functional decline [1–3]. Recent studies have reported that higher PA in the transition from mid to older age reduces the risk of adverse health outcomes in later life [4–7]. This is important because the worldwide economic burden of physical inactivity is significant; inactivity cost healthcare systems $53.8 billion in 2013 [8], and the cost of inaction on physical inactivity reaches $47.6 billion annually [9]. Furthermore, as the risks of physical inactivity [10], multimorbidity [11], and dementia [12] are higher in women than in men, developing public health strategies to promote women’s PA during midlife is vital to reducing gender inequity and improving population health.

Many previous studies have investigated the correlates and determinants of PA in various populations, and have identified individual demographic, psychological, social, and environmental factors that affect PA levels [13–16]. Although some socioeconomic factors are common determinants of PA in men and women [17], other sociodemographic factors, such as living with children, may affect men and women differently, due to socially structured gender roles [18, 19]. Most of the documented determinants of PA relate to the availability of ‘free time’ (e.g., paid work and caring responsibilities) as well as motivation and ability to access activity opportunities, which reflect an array of factors relating to socioeconomic status, education, social support, living and working environments, and health [14]. In this mid-age cohort, menopause, which is known to affect health [20], may also affect PA.

Understanding these determinants during mid-age is important for developing interventions that will help women maintain PA levels at this life stage, and keep them above the disability threshold as they age [21]. However, most studies have measured PA at a single time point [22, 23], even though baseline measures of some sociodemographic factors, health indicators, and health behaviours may change over time [24]. For example, there may be changes in marital status, work hours, and provision of care for children and other family members, all of which impact discretionary time, and are associated with levels of PA [19, 25]. In mid-age, increasing body mass index (BMI) and declining physical and mental health may also be associated with changing patterns of PA [25, 26].

Previous research with women has shown that at the group level, PA increases in mid-age and rapidly declines in older age [27, 28]. However, at the individual level, there may be different PA trajectories at this life stage, which may be influenced by socioeconomic and health characteristics. Although data from the British Regional Heart study have been used to show trajectories of PA in British men across 20 years [29], little is known about individual trajectories of PA in women during the transition from mid-age to early old age. To our knowledge, no previous studies have examined associations between changing sociodemographic and health factors, and trajectories of PA in mid-older women.

Therefore, in this study, we used 21 years of data from a nationally representative cohort of Australian women born in 1946–51 to identify: (1) different PA trajectories in the transition from mid- to early old age; and (2) associations of sociodemographic, behavioural and health factors, and changes in these, with the different PA trajectories.

## Methods

### Study participants

We used data from the 1946–51 cohort of the Australian Longitudinal Study on Women’s Health (ALSWH), an ongoing prospective study [30]. Participants were randomly selected from women born in 1946–51 who were registered with Australia’s universal health insurance system (Medicare), which helps to pay for out-of-hospital health services. The Medicare database includes almost all Australian citizens and some temporary residents [31].

The baseline survey was mailed in 1996 (Survey 1) when participants were 45–50 years (*N* = 13,714), and the recruited participants were generally representative of women the same age in the Australian population [31, 32]. Eight follow-up surveys were mailed at three-year intervals from 1998 (age 47–52) to 2019 (age 68–73). Further details of recruitment methods, response rates and data collection have been described elsewhere [30], and full details of the study can be found at http://www.alswh.org.au. All participants consented to join the ALSWH study, which has ethical approval from the Human Research Ethics Committees (HRECs) of the Universities of Newcastle and Queensland (approval number 2004000224).

Because the PA measure used in 1996 (Survey 1) was different from those in the following surveys, and to minimise bias in identifying trajectories, only data from women who were enrolled in the cohort in 1996 and then completed at least four additional follow-up surveys from 1998 to 2019 were included in the analyses (*N* = 10,371).

### Physical activity

PA was assessed using the modified self-administered version of the Active Australia Survey, which has acceptable reliability and validity among mid-aged women [33]. In each survey, participants reported time spent in the previous week in walking briskly (‘for recreation or exercise or to get to or from places’), moderate leisure-time physical activities (‘like social tennis, moderate exercise classes, recreational swimming, dancing’), and vigorous leisure-time activities (‘like aerobics, competitive sport, vigorous cycling, running, swimming’). Minutes per week spent in each activity were multiplied by a metabolic equivalent (MET) score (3.33 for walking and moderate intensity activity and 6.66 for vigorous activity), and MET.minutes/week were summed to provide total weekly PA. A threshold of 500 MET.minutes/week was used to define meeting the lower range of the current PA guidelines [1, 34]. This is equivalent to 150 min at 3.33 METs or 75 min at 6.66 METs, or various combinations of walking, moderate, and vigorous activity, as described in the 2014 report on the revision of Australia's Physical Activity Guidelines [34]. Outliers (defined as > 3321.7 MET.minutes/week [1.5 times the interquartile range [IQR] above the 75th percentile]) were truncated to 3321.7 MET.minutes/week.

### Potential determinants of different PA trajectories

Since a wide range of factors influence PA levels [13–16], and some of these change over time [24], we selected sociodemographic, behavioural and health characteristics at age 47–52, which have been shown in previous studies to have significant associations with PA [14, 19, 25], and changes in the variables from age 47–52 to 56–61, as the potential determinants of PA trajectories.

### Sociodemographic, behavioural and health characteristics at age 47–52

Sociodemographic, behavioural and health characteristics were reported and measured at age 47–52 and categorised as shown in Table 1. The complete list of variables is shown in Supplementary table 1. Sociodemographic characteristics included country of birth, area of residence, educational attainment, marital status, ability to manage on income, weekly paid work hours, living with children age < 18, providing care for grandchildren, and providing care for someone with long-term illness, disability, or frailty. Household income was not included because its missing rate was high (27.9%). Instead, ability to manage on income (missing rate: 6.8%), which is correlated with PA, was used as a proxy for financial stability [35]. Menopause status was determined using questions about hysterectomy, oophorectomy, hormone treatment, and menstrual pattern [36], and categorised as shown in Table 1. We used reported height and weight to calculate BMI [37], which was categorised as underweight (< 18.5 kg/m^2^), normal weight (18.5–24.9 kg/m^2^), overweight (25.0–29.9 kg/m^2^), or obese (≥ 30.0 kg/m^2^) following the WHO recommendation [38]. Physical health (PH) and mental health (MH) were assessed using the component summary scores from the SF-36 questionnaire, categorised in quartiles [39, 40]. Number of chronic conditions was from a list which included: type 2 diabetes, heart disease, hypertension, stroke, low iron level, asthma, bronchitis/emphysema, osteoporosis, breast cancer, cervical cancer, skin cancer, depression, and anxiety, categorised as 0, 1, 2 or ≥ 3. Smoking and alcohol habits, based on frequency and quantity of tobacco use and alcohol consumption, and frequency of short-term risk drinking [41], were categorised as shown in Table 1.

**Table 1:** Characteristics of analytic and non-analytic samples (Australian women age 47–52)

	**Analytic sample *N* = 10371**^**a**^	**Non-analytic sample *N* = 3343**^**a**^	***p*-value**^**b**^
	Mean (SD)	Mean (SD)	
**Age**	49.1 (1.5)	49.1 (1.5)	0.51
Missing	1	1	
	n (%)	n (%)	
**Country of birth**			
Australia	7957 (77.5%)	2348 (71.6%)	< 0.001
Other English-speaking country	1422 (13.9%)	398 (12.1%)	
Europe	591 (5.8%)	302 (9.2%)	
Asia/Other	291 (2.8%)	231 (7.0%)	
Missing	110	64	
**Area of residence**			
Major city	3299 (33.1%)	782 (33.9%)	0.006
Inner regional	4094 (41.1%)	864 (37.4%)	
Outer regional	2073 (20.8%)	538 (23.3%)	
Remote/Very remote	498 (5.0%)	124 (5.4%)	
Missing	407	1035	
**Educational attainment**			
< 12 years school	4814 (46.8%)	1984 (60.4%)	< 0.001
12 years school	1735 (16.9%)	551 (16.8%)	
Post-school certificate	2118 (20.6%)	481 (14.6%)	
Degree/Higher degree	1623 (15.8%)	269 (8.2%)	
Missing	81	58	
**Marital status**			
Single	312 (3.1%)	88 (3.8%)	< 0.001
Married	8318 (83.5%)	1806 (78.6%)	
Separated/Divorced/Widowed	1328 (13.3%)	404 (17.6%)	
Missing	413	1045	
**Ability to manage on income**			
Easy	1534 (15.9%)	212 (11.5%)	< 0.001
Not too bad	4018 (41.6%)	626 (33.9%)	
Difficult sometimes	2774 (28.7%)	604 (32.7%)	
Impossible/Difficult always	1324 (13.7%)	406 (22.0%)	
Missing	721	1495	
**Hours paid work**			
None	1903 (21.2%)	561 (33.3%)	< 0.001
1–34 h/week	3330 (37.2%)	561 (33.3%)	
≥ 35 h/week	3723 (41.6%)	563 (33.4%)	
Missing	1415	1658	
**Living with children age < 18**			
No	6539 (68.5%)	1247 (68.4%)	0.91
Yes	3006 (31.5%)	577 (31.6%)	
Missing	826	1519	
**Provides care for grandchildren**			
None	5641 (59.1%)	1023 (56.4%)	0.003
Occasionally	3158 (33.1%)	608 (33.5%)	
Weekly/Daily	741 (7.8%)	182 (10.0%)	
Missing	831	1530	
**Provides care for others**			
None	5686 (68.2%)	1043 (70.5%)	0.18
Occasionally	706 (8.5%)	110 (7.4%)	
Weekly/Daily	1941 (23.3%)	326 (22.0%)	
Missing	2038	1864	
**Menopause status**			
Post-menopause	1123 (11.1%)	460 (17.1%)	
HRT/OCP use	1540 (15.3%)	291 (10.8%)	
Pre-menopause	2379 (23.6%)	417 (15.5%)	
Peri-menopause	2461 (24.4%)	539 (20.0%)	
Missing	277	654	
**BMI**			
Underweight/Normal	4479 (49.0%)	746 (44.6%)	< 0.001
Overweight	2873 (31.5%)	520 (31.1%)	
Obese	1782 (19.5%)	406 (24.3%)	
Missing	1237	1671	
**Physical health**^**c**^			
First quartile (highest)	2509 (25.4%)	520 (23.1%)	< 0.001
Second quartile	2560 (26.0%)	467 (20.8%)	
Third quartile	2494 (25.3%)	535 (23.8%)	
Fourth quartile (lowest)	2302 (23.3%)	727 (32.3%)	
Missing	506	1094	
**Mental health**^**c**^			
First quartile (highest)	2528 (25.6%)	498 (22.1%)	< 0.001
Second quartile	2545 (25.8%)	486 (21.6%)	
Third quartile	2433 (24.7%)	595 (26.5%)	
Fourth quartile (lowest)	2359 (23.9%)	670 (29.8%)	
Missing	506	1094	
**Chronic conditions**			
0	3545 (35.4%)	802 (34.5%)	< 0.001
1	3197 (31.9%)	636 (27.4%)	
2	1858 (18.6%)	431 (18.6%)	
≥ 3	1415 (14.1%)	454 (19.5%)	
Missing	356	1020	
**Smoking status**			
Non-smoker	5532 (57.2%)	975 (51.7%)	< 0.001
Ex-smoker	2633 (27.2%)	438 (23.2%)	
Current smoker	1513 (15.6%)	473 (25.1%)	
Missing	693	1457	
**Alcohol status**			
Non-drinker	1187 (12.3%)	355 (18.9%)	< 0.001
Rare drinker	2681 (27.7%)	627 (33.4%)	
Low-risk drinker^d^	5265 (54.4%)	786 (41.9%)	
Risky/high-risk drinker^d^	537 (5.6%)	108 (5.8%)	
Missing	701	1467	

*HRT* Hormone replacement therapy, *OCP* Oral contraceptive pill

Percentage for each category was calculated using data after excluding missing values

^a^Continuous variable: mean (SD); categorical variables: n (%); missing values: only n

^b^Continuous variable: Wilcoxon rank sum test; categorical variables: Chi-squared test

^c^Physical and mental component summary scores from SF36

^d^Low-risk drinker, up to 14 drinks per week; Risky/High-risk drinker, 15 or more drinks per week

*Changes in sociodemographic, behavioural and health characteristics from Survey 2 (age 47*–52*) to Survey 5 (age 56–61).*

Variables indicating changes in area of residence, marital status, ability to manage on income, paid work hours, living with children age < 18, provision of care for grandchildren or someone requiring care, BMI, PH, MH, smoking status, and alcohol status, from age 47–52 (Survey 2, 1998) to 56–61 (Survey 5, 2007) were created and categorised as shown in Tables 3 and 5.

### Statistical analysis

#### Physical activity trajectories

Group-based trajectory modelling was used to identify clusters of women who followed a similar trajectory of PA over 21 years using the STATA plugin traj [42]. The number of groups (2–5) and polynomials (linear, quadratic, cubic) were determined based on goodness-of-fit of the model. The best-fit model was selected using Bayesian information criterion (BIC), Akaike information criterion (AIC), and log-likelihood. The adequacy of the model was confirmed based on the following criteria: posterior probabilities of group membership (> 0.7 probability of an individual belonging to each of the trajectory groups); the odds of correct classification > 5 [43]. Full information maximum likelihood estimation was used for the group-based trajectory modelling to account for missing data, assuming these were missing at random.

### Determinants of different physical activity trajectories

We conducted analyses to elucidate the trajectories of (a) probability of meeting the PA recommendation (≥ 500 MET.minutes/week) [1, 34], and (b) total PA (MET.minutes/week). However, as the adequacy of the model examining trajectories of meeting guidelines (a) was not acceptable (due to poor posterior probabilities, < 0.7) (Supplementary Fig. 1 and Supplementary table 2), we did not conduct analyses to examine the determinants of meeting guidelines.

Multinomial logistic regression models were conducted to investigate the association of sociodemographic, behavioural and health characteristics at age 47–52, and changes in selected variables from age 47–52 to 56–61, with PA trajectories. For this, the R package nnet was used [44]. The models were adjusted for age and PA trajectory was the dependent variable. Independent variables were (1) sociodemographic factors, health indicators and health behaviours at age 47–52, and (2) changes in explanatory factors from age 47–52 to 56–61. These variables were included using a hierarchical model in two blocks, with variables from the first block retained for the next stage if the *p*-value was ≤ 0.20. Each odds ratio (OR) was calculated by dividing the odds of being in one trajectory group (e.g., Low-stable, compared with High-stable) in each category of the exposure variable (e.g., higher degree) by the odds of being in these groups (e.g., Low-stable vs High-stable) in the referent category (e.g., < 12 years education) of the exposure variable. ORs < 1/ > 1 indicate that the participants in one category of the exposure group (i.e., higher degree) are, respectively, less likely/more likely than the reference group (e.g., < 12 years of education) to be in each trajectory group, compared with the High-stable group.

The proportion of participants with missing values in the full model, which included sociodemographic, behavioural and health characteristics at age 47–52 and changes in variables from age 47–52 to 56–61 was 41.4%; this was largely due to failure to respond to one complete survey. As missing at random (MAR) is likely in many situations [45], we used a multiple imputation procedure which handles not only MAR but also missing completely at random and missing not at random [45], and conducted multiple imputations, using the R package MICE [46]. Fifty data sets were created, and the combined ORs and 95% confidence intervals (CI) of each data set were used to obtain the estimates.

Group-based trajectory modelling was carried out using STATA 16.0 (StataCorp, Texas, USA). Multinomial logistic regression models and multiple imputation were conducted using R 3.6.3 (R Foundation for Statistical Computing, Vienna, Austria).

## Results

Of the 13,714 women in the original cohort, data from 10,371 (75.6%) were included in this analysis; 6,969 participants provided valid responses to all surveys. The comparison of characteristics at age 47–52 of the analytic and non-analytic samples is shown in Table 1. Because of the large sample size, there were small but statistically significant differences in most characteristics (except age, living with children, and providing care for someone requiring care) between women whose data were and were not included in the analyses. However, with the exception of education, there were no meaningful differences in the characteristics of the women in analytic and non-analytic samples (Table 1).

**Fig. 1:**
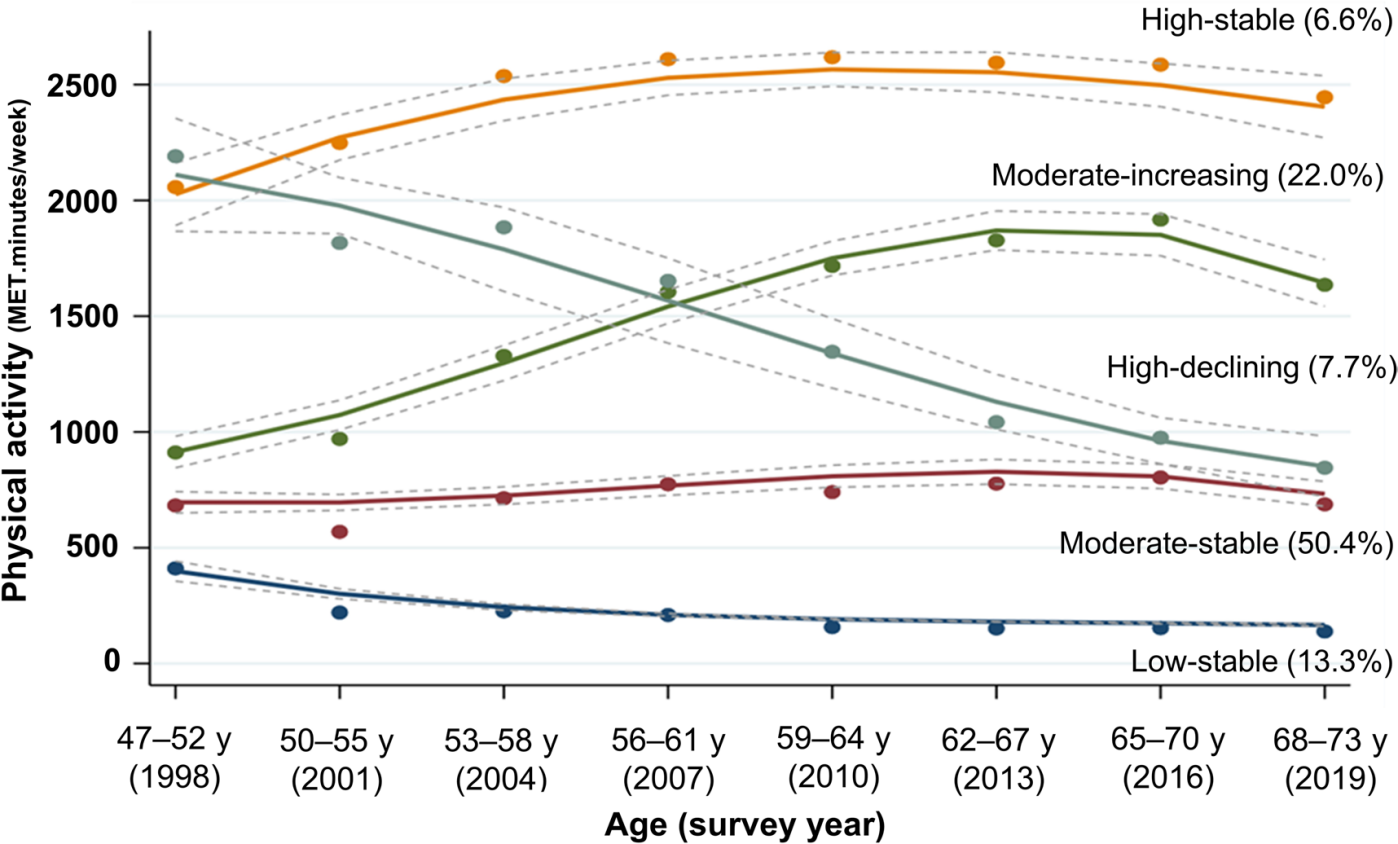
Physical activity trajectories among mid-aged women (MET.minutes/week) (*N* = 10,371) Group-based trajectory models. Outliers (defined as > 3321.7 MET.minutes/week [1.5 times the interquartile range above the 75th percentile]) were truncated to 3321.7 MET.minutes/week

PA trajectories from age 47–52 to 68–73 are shown in Fig. 1. Model fitting and adequacy are shown in Supplementary table 2. We selected the five trajectories of the cubic polynomials model, which met the adequacy criteria; posterior probabilities ranged from 0.741 to 0.865, and the odds of correct classification ranged from 5.32 to 90.25 (Supplementary table 3). PA trajectories were labelled Low-stable (13.3% of participants), Moderate-stable (50.4%), Moderate-increasing (22.2%), High-declining (7.7%), and High-stable (6.6%) (Fig. 1).

**Fig. 2:**
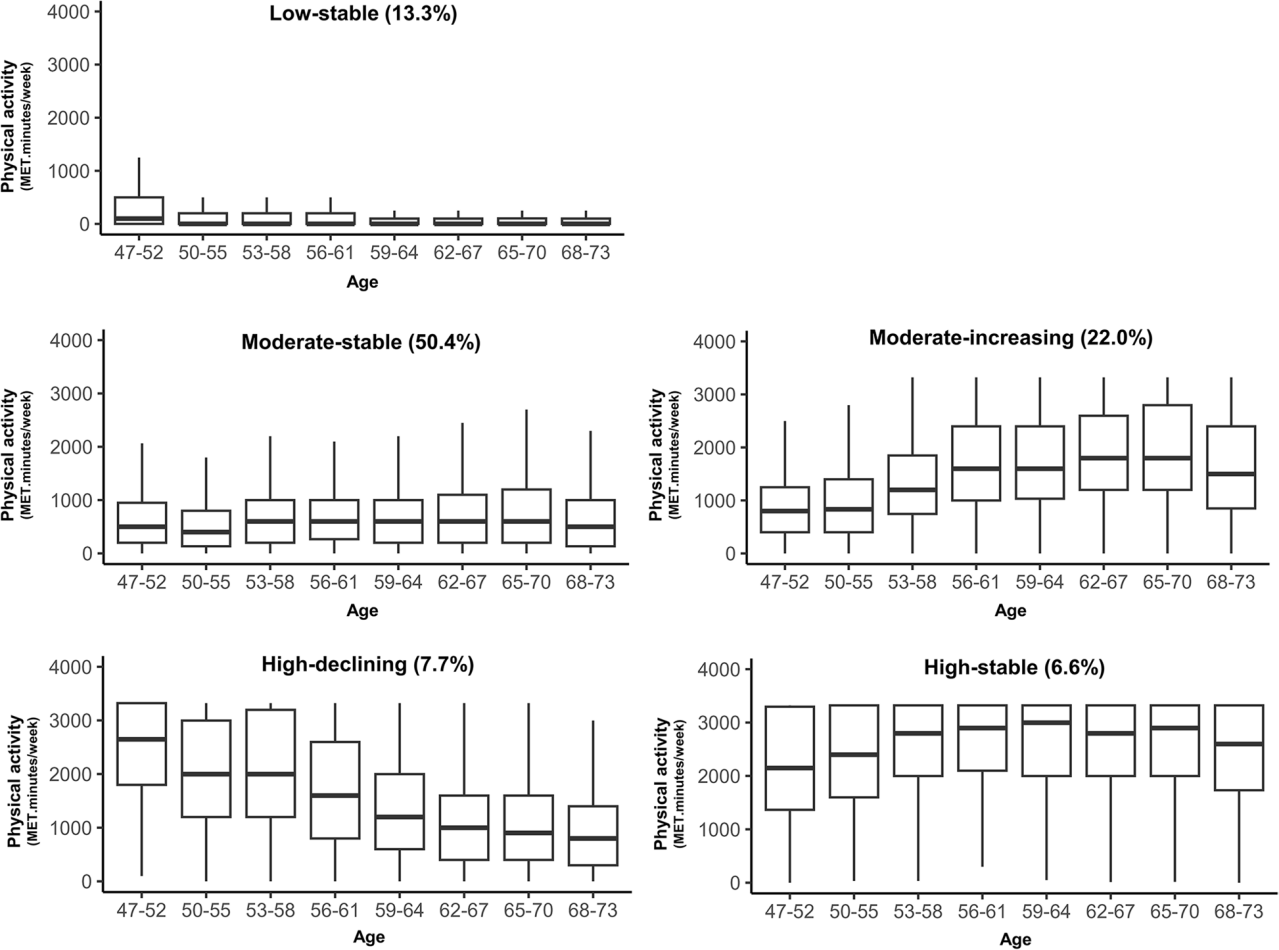
Physical activity at each survey for each of the PA trajectories (*N* = 10,371) Outliers (defined as > 3321.7 MET.minutes/week [1.5 times the interquartile range above the 75th percentile]) were truncated to 3321.7 MET.minutes/week

Box plots in Fig. 2 are used to show the medians and IQRs of PA at each survey for each of the PA trajectories, and the data are shown in Table 2. Medians of PA values in the Low-, Moderate-, and High-stable trajectory groups were fairly consistent over time. In the Low-stable group, median PA was zero at every survey from age 50–55. In the Moderate-stable group, median PA was between 400 and 600 MET.minutes/week at each survey. Median PA in the High-stable group was > 2000 MET.minutes/week at every survey and close to 3000 MET.minutes/week at age 59–64.

**Table 2:** Physical activity (MET.minutes/week) at each survey for each of the physical activity trajectories (*N* = 10,371)

	**Low-stable**	**Moderate-stable**	**Moderate-increasing**	**High-declining**	**High-stable**
	**(13.3%)**	**(50.4%)**	**(22.0%)**	**(7.7%)**	**(6.6%)**
	**Median (IQR)**	**Median (IQR)**	**Median (IQR)**	**Median (IQR)**	**Median (IQR)**
47–52 y	99.9 (0, 499.5)	499.5 (199.8, 949.0)	799.2 (399.6, 1265.4)	2647.3 (1798.2, 3321.7)	2147.8 (1365.3, 3296.7)
50–55 y	0 (0, 199.8)	399.6 (133.2, 799.2)	832.5 (399.6, 1398.6)	1998.0 (1198.8, 2997.0)	2397.6 (1598.4, 3321.7)
53–58 y	0 (0, 199.8)	599.4 (199.8, 999.0)	1198.8 (732.6, 1831.5)	1998.0 (1198.8, 3196.8)	2797.2 (1998.0, 3321.7)
56–61 y	0 (0, 199.8)	599.4 (266.4, 999.0)	1598.4 (999.0, 2397.6)	1598.4 (799.2, 2597.4)	2897.1 (2097.9, 3321.7)
59–64 y	0 (0, 116.6)	599.4 (199.8, 999.0)	1598.4 (1032.3, 2397.6)	1198.8 (599.4, 1998.0)	2997.0 (1998.0, 3321.7)
62–67 y	0 (0, 199.8)	599.4 (199.8, 1098.9)	1798.2 (1198.8, 2597.4)	999.0 (399.6, 1598.4)	2797.2 (1998.0, 3321.7)
65–70 y	0 (0, 199.8)	599.4 (199.8, 1198.8)	1798.2 (1198.8, 2797.2)	899.1 (399.6, 1536.0)	2797.2 (1998.0, 3321.7)
68–73 y	0 (0, 199.8)	499.5 (149.8, 999.0)	1431.9 (799.2, 2347.7)	799.2 (299.7, 1398.6)	2597.4 (1665.0, 3321.7)
Overall^a^	183.2 (99.9, 274.7)	705.5 (507.8, 912.6)	1482.9 (1291.4, 1717.0)	1554.7 (1339.3, 1835.7)	2425.7 (2213.4, 2694.2)

*IQR *Interquartile range, *y *year

^a^Median of 21-year overall physical activity was calculated by adding median MET.minutes/week for physical activity at each survey and dividing by 8 (number of surveys from age 47–52 to 68–73)

In contrast with the three ‘stable’ trajectories (Low, Moderate, and High), median PA in the High-declining group declined markedly, from 2647 MET.minutes/week at 47–52 years to 799 MET.minutes/week at age 68–73, while in the Moderate-increasing group, PA increased from 799 MET.minutes/week at age 47–52 to a high of 1798 MET.minutes/week at age 62–70 years (Fig. 2 and Table 2).

Characteristics of the participants in each trajectory group are shown in Table 3, and the ORs for being in each group, compared with the High-stable PA group, are shown in Tables 4 (characteristics at age 47–52) and 5 (changes in explanatory variables). As expected, demographic characteristics, such as education, ability to manage on income, and living with children, varied across the Low, Moderate and High trajectory groups. For example, women with any post-school education were less likely to be in the Low-stable trajectory group, and those with a university degree were also less likely to be in the High-declining group (than the High-stable group). When compared with those in the High-stable group, women who reported always having difficulty managing on income were over-represented in every group, and those who were living with children were more likely to be in the Moderate-stable or Moderate-increase trajectory groups. Women in paid work, and those who were post-menopause, were less likely to be in the High-declining trajectory group, and those who lived in an outer regional area, and provided care for grandchildren (occasionally) were more likely to be in the High-declining group (than the High-stable group).

**Table 3:** Characteristics of participants in each physical activity trajectory (*N* = 10,371)

	**Low-stable (13.3%)**	**Moderate-stable (50.4%)**	**Moderate-increasing (22.0%)**	**High-declining (7.7%)**	**High-stable (6.6%)**
**Characteristics at age 47–52**					
**Age – mean (SD)**	49.1 (1.4)	49.0 (1.6)	49.0 (1.8)	49.2 (1.5)	49.2 (1.4)
**Country of birth**					
Australia	80.0	77.5	76.4	78.2	76.3
Other English-speaking country	10.9	13.7	15.9	13.6	14.5
Europe	6.5	5.6	5.4	6.2	6.2
Asia/Other	2.7	3.2	2.2	2.0	3.0
**Area of residence**					
Major city	30.1	34.1	33.3	26.8	34.7
Inner regional	40.5	40.1	42.6	43.8	42.3
Outer regional	23.7	20.7	19.4	24.1	18.3
Remote/Very remote	5.6	5.1	4.8	5.2	4.7
**Educational attainment**					
< 12 years school	63.6	46.5	40.0	47.4	39.1
12 years school	14.4	17.0	16.9	20.7	16.0
Post-school certificate	14.3	20.4	23.5	20.0	25.2
Degree/Higher degree	7.7	16.1	19.7	12.0	19.7
**Marital status**					
Single	4.5	2.9	2.9	3.5	3.3
Married	79.9	84.2	83.8	83.6	82.7
Separated/Divorced/Widowed	15.6	12.9	13.3	13.0	14.0
**Ability to manage on income**					
Easy	9.6	14.8	19.5	16.9	22.9
Not too bad	35.3	41.9	43.6	40.7	43.9
Difficult sometimes	32.2	29.6	25.9	29.1	26.0
Impossible/Difficult always	22.9	13.7	11.0	13.3	7.2
**Hours paid work**					
None	31.6	21.2	16.5	28.9	17.6
1–34 h/week	31.9	37.3	39.4	39.0	39.3
≥ 35 h/week	36.6	41.5	44.1	32.2	43.1
**Living with children age < 18**					
No	71.6	66.7	68.6	72.0	72.9
Yes	28.4	33.3	31.4	28.0	27.1
**Provides care for grandchildren**					
None	55.5	59.0	61.6	55.5	62.2
Occasionally	35.5	33.3	31.2	34.0	32.3
Weekly/Daily	9.1	7.7	7.2	10.6	5.5
**Provision of care for others**					
None	68.6	68.5	69.1	63.9	68.4
Occasionally	8.3	8.2	8.9	10.7	7.1
Weekly/Daily	23.2	23.2	22.1	25.4	24.6
**Menopause status**					
Peri-menopause	22.9	24.8	25.1	25.8	24.4
Surgical menopause	33.0	24.3	23.7	24.9	20.5
HRT/OCP use	14.9	15.8	14.9	16.5	13.3
Pre-menopause	18.2	24.2	25.2	23.1	27.7
Post-menopause	11.0	10.9	11.1	9.7	14.0
**BMI**					
Underweight/Normal	33.0	46.3	56.7	52.5	67.4
Overweight	30.9	33.4	29.2	30.9	25.4
Obese	36.1	20.2	14.1	16.6	7.2
**Physical health**^**a**^					
First quartile (highest)	15.3	23	30.5	26.7	39.8
Second quartile	17.4	24.8	27.7	26.2	30.2
Third quartile	24.5	26.2	23.7	26.9	18.9
Fourth quartile (lowest)	42.9	26	18.1	20.1	11.1
**Mental health**^**a**^					
First quartile (highest)	19.1	23.6	27.9	31.4	31.4
Second quartile	19.9	25.0	27.6	21.8	29.6
Third quartile	26.0	25.4	24.4	24.3	22.2
Fourth quartile (lowest)	34.9	26.0	20.1	22.5	16.8
**Chronic conditions**					
0	28.3	34.5	39.1	38.9	40.3
1	28.6	31.9	33.6	31.2	33.5
2	23.4	18.9	16.7	16.3	15.0
≥ 3	19.8	14.7	10.6	13.5	11.1
**Smoking status**					
Non-smoker	48.3	58.3	59.2	55.4	56.8
Ex-smoker	24.4	27.1	28.5	26.8	30.7
Current smoker	27.2	14.6	12.2	17.8	12.5
**Alcohol status**^**a**^					
Non-drinker	16.3	12.5	10.7	10.7	9.2
Rare drinker	33.1	29.1	24.1	24.7	21.7
Low-risk drinker^a^	42.5	53.4	60.1	58.3	63.7
Risky/high-risk drinker^a^	8.0	5.1	5.1	6.3	5.4
**Changes from age 47–52 to age 56–61**					
**Area of residence**					
No change	90.8	90.2	89.4	90.2	89.7
Country–city	5.9	6.3	6.6	7.0	5.8
City–country	3.3	3.5	4.0	2.8	4.5
**Marital status**					
No change	88.1	89.2	88.7	88.5	90.5
Not–married	3.4	3.1	3.8	3.0	2.4
Married–not	8.5	7.7	7.5	8.5	7.1
**Ability to manage on income**					
No change	71.3	69.8	72.6	69.9	73.8
Difficult–easy	16.1	19.2	18.1	17.5	16.6
Easy–difficult	12.7	11.1	9.4	12.7	9.7
**Employment status**					
No change	56.2	54.3	52.5	55.0	51.1
Worked–no work	30.3	29.5	31.7	24.6	32.8
No work–worked	13.5	16.2	15.8	20.3	16.0
**Living with children age < 18**					
No change	72.6	68.5	70.3	72.1	73.5
Lived with–not	24.9	30.1	28.9	25.6	25.2
Not–lived with	2.4	1.4	0.8	2.3	1.3
**Provides care for grandchildren**					
No change	52.2	50.7	51.2	50.0	53.0
Decreased	16.8	15.9	15.0	15.9	13.8
Increased	31.1	33.5	33.8	34.1	33.2
**Provides care for others**					
No change	60.7	61.4	61.1	58.4	61.0
Decreased	20.1	19.4	18.9	23.0	19.7
Increased	19.3	19.2	20.0	18.6	19.3
**BMI**					
No change	85.6	82.6	83.5	81.3	84.3
Normal/overweight–obese	11.8	13.8	12.4	15.0	10.6
Obese–normal/overweight	2.6	3.6	4.1	3.6	5.1
**Physical health**^**b**^					
No change	46.3	37.1	39.0	35.7	41.0
Lower–higher	31.2	42.1	44.1	43.6	40.2
Higher–lower	22.4	20.8	17.0	20.6	18.8
**Mental health**^**b**^					
No change	42.6	41.2	43.4	41.2	41.1
Lower–higher	27.6	29.2	28.0	27.6	32.6
Higher–lower	29.8	29.5	28.6	31.1	26.4
**Smoking status**					
No change	90.1	91.5	91.7	92.1	93.0
Not–smoking	1.1	0.9	1.0	0.8	0.7
Smoking–not	8.8	7.6	7.3	7.1	6.3
**Alcohol status**					
No change	68.0	72.2	75.0	72.1	75.6
Increased	15.6	16.2	15.2	13.8	14.6
Decreased	16.4	11.6	9.8	14.0	9.8

Mean (SD); %

*h* hours, *BMI* Body mass index, *HRT* Hormone replacement therapy, *OCP* Oral contraceptive

^a^Low-risk drinker, up to 14 drinks per week; Risky/High-risk drinker, more than 15 drinks per week

^b^Physical and mental health were categorised as higher and lower based on the median, and combinations at age 47–52 and 56–61 (higher–higher, higher–lower, lower–higher, and lower–lower) were created. No change category includes higher–higher and lower–lower

**Table 4:** Characteristics at age 47–52 and physical activity trajectories (Reference: High-stable) (*N* = 10,371)

	**Low-stable**	**Moderate-stable**	**Moderate-increasing**	**High-declining**
	**Adjusted OR**	**Adjusted OR**	**Adjusted OR**	**Adjusted OR**
	**(95% CI)**	**(95% CI)**	**(95% CI)**	**(95% CI)**
**Characteristics at age 47–52**				
Australia	Reference	Reference	Reference	Reference
Other English-speaking country	0.92 (0.68, 1.25)	1.07 (0.84, 1.36)	1.19 (0.92, 1.54)	1.08 (0.78, 1.48)
Europe	1.00 (0.66, 1.52)	0.90 (0.63, 1.28)	0.89 (0.61, 1.31)	1.02 (0.65, 1.62)
Asia/Other	1.36 (0.73, 2.51)	1.07 (0.64, 1.78)	0.70 (0.40, 1.23)	0.88 (0.43, 1.83)
**Area of residence**				
Major city	Reference	Reference	Reference	Reference
Inner regional	0.93 (0.73, 1.18)	0.89 (0.73, 1.08)	1.01 (0.82, 1.25)	1.24 (0.95, 1.62)
Outer regional	1.18 (0.88, 1.58)	1.01 (0.79, 1.29)	1.03 (0.79, 1.33)	**1.46 (1.06, 2.00)**
Remote/Very remote	1.15 (0.71, 1.88)	1.03 (0.68, 1.56)	1.01 (0.65, 1.57)	1.32 (0.77, 2.26)
**Educational attainment**				
< 12 years school	Reference	Reference	Reference	Reference
12 years school	**0.64 (0.48, 0.85)**	0.96 (0.75, 1.23)	1.06 (0.82, 1.38)	1.20 (0.88, 1.64)
Post-school certificate	**0.44 (0.33, 0.57)**	**0.75 (0.60, 0.93)**	0.94 (0.75, 1.19)	0.76 (0.57, 1.02)
Degree/Higher degree	**0.35 (0.25, 0.48)**	0.79 (0.62, 1.02)	1.04 (0.80, 1.35)	**0.68 (0.48, 0.97)**
**Marital status**				
Married	Reference	Reference	Reference	Reference
Single	1.29 (0.74, 2.25)	0.83 (0.51, 1.35)	0.86 (0.51, 1.44)	1.25 (0.68, 2.33)
Separated/Divorced/Widowed	0.87 (0.64, 1.18)	0.81 (0.63, 1.05)	0.91 (0.69, 1.19)	0.91 (0.65, 1.28)
**Ability to manage on income**				
Easy	Reference	Reference	Reference	Reference
Not too bad	**1.39 (1.03, 1.89)**	**1.26 (1.00, 1.58)**	1.09 (0.86, 1.39)	1.09 (0.80, 1.48)
Difficult sometimes	**1.56 (1.12, 2.18)**	**1.30 (1.01, 1.68)**	1.04 (0.79, 1.37)	1.18 (0.84, 1.67)
Impossible/Difficult always	**2.74 (1.76, 4.26)**	**1.84 (1.25, 2.69)**	**1.51 (1.01, 2.26)**	1.58 (0.99, 2.52)
**Hours paid work**				
None	Reference	Reference	Reference	Reference
1–34 h/week	0.80 (0.59, 1.08)	0.99 (0.76, 1.29)	1.15 (0.87, 1.53)	**0.73 (0.53, 1.00)**
≥ 35 h/week	1.02 (0.76, 1.38)	1.15 (0.88, 1.49)	1.26 (0.95, 1.65)	**0.61 (0.44, 0.84)**
**Living with children age < 18**				
No	Reference	Reference	Reference	Reference
Yes	1.24 (0.97, 1.58)	**1.38 (1.13, 1.69)**	**1.23 (1.00, 1.53)**	1.15 (0.88, 1.50)
**Provides care for grandchildren**				
None	Reference	Reference	Reference	Reference
Occasionally	1.06 (0.69, 1.63)	1.11 (0.78, 1.59)	1.22 (0.84, 1.77)	**1.58 (1.03, 2.43)**
Weekly/Daily	0.81 (0.62, 1.06)	0.88 (0.70, 1.10)	0.88 (0.69, 1.11)	1.02 (0.76, 1.37)
**Provides care for others**				
None	Reference	Reference	Reference	Reference
Occasionally	0.88 (0.70, 1.10)	0.95 (0.78, 1.14)	0.92 (0.75, 1.13)	0.98 (0.76, 1.26)
Weekly/Daily	0.99 (0.64, 1.52)	1.14 (0.78, 1.68)	1.23 (0.82, 1.84)	1.57 (0.99, 2.48)
**Menopause status**				
Peri-menopause	Reference	Reference	Reference	Reference
Surgical menopause	1.07 (0.80, 1.43)	0.94 (0.73, 1.21)	1.06 (0.81, 1.38)	0.93 (0.67, 1.28)
HRT/OCP use	1.17 (0.83, 1.64)	1.19 (0.90, 1.59)	1.12 (0.83, 1.51)	1.17 (0.82, 1.69)
Pre-menopause	0.83 (0.62, 1.11)	0.91 (0.72, 1.16)	0.88 (0.69, 1.13)	0.84 (0.61, 1.14)
Post-menopause	0.83 (0.58, 1.17)	0.79 (0.59, 1.05)	0.82 (0.61, 1.12)	**0.64 (0.43, 0.95)**
**BMI**				
Underweight/Normal	Reference	Reference	Reference	Reference
Overweight	**2.16 (1.70, 2.76)**	**1.76 (1.45, 2.15)**	**1.31 (1.05, 1.62)**	**1.43 (1.10, 1.85)**
Obese	**6.47 (4.54, 9.21)**	**3.16 (2.28, 4.39)**	**2.07 (1.47, 2.93)**	**2.38 (1.62, 3.51)**
**Physical health**				
First quartile (highest)	Reference	Reference	Reference	Reference
Second quartile	**1.71 (1.28, 2.28)**	**1.60 (1.29, 1.99)**	**1.28 (1.01, 1.61)**	1.32 (0.99, 1.78)
Third quartile	**3.01 (2.22, 4.08)**	**2.36 (1.85, 3.01)**	**1.66 (1.28, 2.15)**	**2.00 (1.46, 2.74)**
Fourth quartile (lowest)	**6.65 (4.75, 9.32)**	**3.61 (2.69, 4.84)**	**2.14 (1.57, 2.91)**	**2.19 (1.52, 3.16)**
**Mental health**				
First quartile (highest)	Reference	Reference	Reference	Reference
Second quartile	1.22 (0.92, 1.62)	1.18 (0.94, 1.48)	1.08 (0.85, 1.37)	0.79 (0.59, 1.07)
Third quartile	**1.91 (1.43, 2.57)**	**1.53 (1.2, 1.95)**	1.27 (0.98, 1.64)	1.15 (0.85, 1.57)
Fourth quartile (lowest)	**2.88 (2.09, 3.96)**	**2.07 (1.58, 2.73)**	**1.42 (1.06, 1.90)**	1.36 (0.96, 1.92)
**Chronic conditions**				
0	Reference	Reference	Reference	Reference
1	1.00 (0.78, 1.28)	0.99 (0.81, 1.20)	0.96 (0.77, 1.18)	0.90 (0.69, 1.17)
2	1.30 (0.96, 1.75)	1.09 (0.84, 1.41)	0.96 (0.72, 1.26)	0.90 (0.65, 1.27)
≥ 3	0.95 (0.67, 1.34)	0.92 (0.68, 1.23)	0.73 (0.53, 1.01)	0.85 (0.58, 1.25)
**Smoking status**				
Non-smoker	Reference	Reference	Reference	Reference
Ex-smoker	0.89 (0.70, 1.13)	0.84 (0.70, 1.03)	0.87 (0.71, 1.07)	0.86 (0.67, 1.11)
Current smoker	**2.22 (1.65, 2.99)**	1.12 (0.85, 1.46)	0.93 (0.69, 1.23)	1.33 (0.96, 1.86)
**Alcohol status**				
Risky/High-risk drinker^a^	Reference	Reference	Reference	Reference
Non-drinker	1.01 (0.61, 1.70)	1.31 (0.82, 2.08)	1.21 (0.74, 1.99)	0.88 (0.49, 1.57)
Rare drinker	0.86 (0.54, 1.36)	1.29 (0.85, 1.94)	1.15 (0.74, 1.78)	0.89 (0.53, 1.50)
Low-risk drinker^a^	**0.58 (0.38, 0.90)**	0.98 (0.67, 1.43)	1.04 (0.69, 1.57)	0.9 (0.56, 1.45)

Multinomial logistic regression models adjusted for age. Reference = High group. ORs = probability of being in each trajectory compared with being in that category in the High group. OR<1 indicates less likely, and OR>1 suggests more likely to be in each trajectory group, compared with the High-stable group

*OR* Odds ratio, *CI* Confidence interval, *h* hours, *BMI* Body mass index, *HRT* Hormone replacement therapy, *OCP* Oral contraceptive

BMI, PH and MH were strong determinants of PA trajectory. Compared with being in the High-stable group, women who were overweight or obese and those in the lower PH and MH quartiles were over-represented in every other trajectory group.

There were few significant associations between changes in these determinants and PA trajectory group (Table 5). Compared with the High-stable group, women who married between age 47–52 and 56–61 were over-represented in the Low-, Moderate-stable and Moderate-increasing groups, while those who reported adverse changes in managing on income (easy to difficult) were more likely to be in the High-declining group. Women who started paid work were less likely to be in the Low-stable group. Women who changed from normal or overweight to obese were more likely to be in the Low-stable, Moderate-stable, and High-declining groups, and those with declining PH and MH were more likely to be in the Low- and Moderate-stable groups. Conversely, those whose health improved were less likely to be in the Low- and Moderate-stable groups (Table 5).

**Table 5:** Changes in explanatory factors and physical activity trajectories (Reference: High-stable) (*N* = 10,371)

	**Low-stable**	**Moderate-stable**	**Moderate-increasing**	**High-declining**
	**Adjusted OR**	**Adjusted OR**	**Adjusted OR**	**Adjusted OR**
	**(95% CI)**	**(95% CI)**	**(95% CI)**	**(95% CI)**
**Changes from age 47–52 to 56–61**				
**Area of residence**				
No change	Reference	Reference	Reference	Reference
Country–city	1.01 (0.68, 1.49)	1.06 (0.76, 1.48)	1.07 (0.75, 1.52)	0.79 (0.51, 1.22)
City–country	0.98 (0.64, 1.51)	1.04 (0.72, 1.50)	0.98 (0.67, 1.45)	0.85 (0.52, 1.37)
**Marital status**				
No change	Reference	Reference	Reference	Reference
Not–married	**2.29 (1.13, 4.65)**	**1.87 (1.02, 3.44)**	**2.02 (1.07, 3.80)**	1.71 (0.78, 3.71)
Married–not	0.85 (0.56, 1.28)	0.9 (0.64, 1.28)	0.97 (0.67, 1.41)	1.00 (0.65, 1.56)
**Ability to manage on income**				
No change	Reference	Reference	Reference	Reference
Difficult–easy	0.89 (0.65, 1.20)	1.08 (0.83, 1.39)	1.08 (0.82, 1.42)	0.86 (0.62, 1.20)
Easy–difficult	1.36 (0.96, 1.93)	1.15 (0.85, 1.55)	0.98 (0.71, 1.34)	**1.46 (1.01, 2.12)**
**Employment status**				
No change	Reference	Reference	Reference	Reference
Worked–no work	0.87 (0.67, 1.13)	0.81 (0.65, 1.00)	0.89 (0.71, 1.12)	0.76 (0.57, 1.02)
No work–worked	**0.61 (0.44, 0.85)**	0.82 (0.62, 1.09)	0.91 (0.67, 1.22)	0.90 (0.64, 1.28)
**Living with children age < 18**				
No change	Reference	Reference	Reference	Reference
Lived with–not	0.56 (0.27, 1.16)	0.72 (0.38, 1.38)	0.87 (0.44, 1.73)	0.87 (0.38, 1.97)
Not–lived with	1.29 (0.55, 3.01)	0.91 (0.42, 1.96)	0.58 (0.24, 1.38)	1.42 (0.58, 3.49)
**Provision of care for grandchildren**				
No change	Reference	Reference	Reference	Reference
Decreased	1.20 (0.83, 1.74)	1.16 (0.85, 1.59)	1.10 (0.79, 1.54)	1.00 (0.67, 1.49)
Increased	0.85 (0.67, 1.09)	1.03 (0.84, 1.25)	1.06 (0.86, 1.31)	1.10 (0.85, 1.42)
**Provision of care for someone requiring care**				
No change	Reference	Reference	Reference	Reference
Decreased	1.28 (0.85, 1.91)	1.11 (0.79, 1.56)	1.05 (0.73, 1.50)	1.20 (0.77, 1.87)
Increased	0.86 (0.65, 1.14)	0.88 (0.69, 1.11)	0.96 (0.75, 1.23)	0.94 (0.68, 1.28)
**BMI**				
No change	Reference	Reference	Reference	Reference
Normal/overweight–obese	**2.17 (1.37, 3.45)**	**1.82 (1.20, 2.77)**	1.37 (0.89, 2.13)	**1.65 (0.99, 2.73)**
Obese–normal/overweight	**0.35 (0.15, 0.78)**	**0.49 (0.24, 1.02)**	0.73 (0.34, 1.58)	0.42 (0.17, 1.08)
**Physical health**^**a**^				
No change	Reference	Reference	Reference	Reference
Lower–higher	**0.38 (0.28, 0.50)**	**0.64 (0.50, 0.82)**	0.79 (0.62, 1.02)	0.82 (0.60, 1.11)
Higher–lower	**1.97 (1.50, 2.59)**	**1.54 (1.24, 1.92)**	1.16 (0.92, 1.47)	1.35 (1.00, 1.81)
**Mental health**^**a**^				
No change	Reference	Reference	Reference	Reference
Lower–higher	**0.63 (0.48, 0.82)**	**0.68 (0.54, 0.85)**	**0.67 (0.52, 0.85)**	0.77 (0.57, 1.04)
Higher–lower	**1.55 (1.17, 2.04)**	**1.34 (1.07, 1.67)**	1.12 (0.89, 1.42)	1.28 (0.96, 1.71)
**Smoking status**				
No change	Reference	Reference	Reference	Reference
Not–smoking	1.74 (0.53, 5.75)	1.32 (0.46, 3.82)	1.57 (0.52, 4.69)	1.32 (0.34, 5.12)
Smoking–not	0.67 (0.41, 1.08)	1.06 (0.69, 1.63)	1.25 (0.79, 1.96)	0.78 (0.46, 1.33)
**Alcohol status**				
No change	Reference	Reference	Reference	Reference
Increased	0.90 (0.65, 1.25)	0.96 (0.72, 1.28)	0.98 (0.72, 1.32)	0.91 (0.63, 1.32)
Decreased	1.15 (0.81, 1.63)	1.02 (0.75, 1.39)	0.95 (0.68, 1.32)	1.20 (0.83, 1.75)

*OR* Odds ratio, *CI* Confidence interval, *h* hours, *BMI* Body mass index, *HRT* Hormone replacement therapy, *OCP* Oral contraceptive

^a^Physical and mental health were categorised as higher and lower based on the median, and combinations at age 47–52 and 56–61 (higher–higher, higher–lower, lower–higher, and lower–lower) were created. No change category includes higher–higher and lower–lower

Multinomial logistic regression models adjusted for age. Reference = High group. ORs = probability of being in each trajectory compared with being in that category in the High group. OR < 1 indicates less likely, and OR > 1 suggests more likely to be in each trajectory group, compared with the High-stable group

## Discussion

Our aims were to identify different PA trajectories in the transition from mid- to early old age, and the associations of sociodemographic and health factors, and changes in these, with the trajectories. Using data which spanned 21 years (when the women were between 47–52 and 68–73 years), we identified five PA distinct trajectories. The majority of women (70%) maintained fairly consistent PA levels over time, but with starkly contrasting median PA of zero (Low-stable), 400–600 MET.minutes/week (Moderate-stable), or 2000–3000 MET.minutes/week (High-stable). PA levels in the Moderate-stable group are commensurate with meeting the lower range of the current PA guidelines (150 min/week of moderate-to-vigorous PA [MVPA] ≈ 500 MET.minutes/week), and in the High-stable group are well above the upper range of the recommendation (300 min/week of MVPA or 1000 MET.minutes/week) [1, 34].

Two trajectories of changing PA were also identified; one small group (8% of the participants) whose initially high PA levels (> 2000 MET.minutes/week) declined markedly over time to around 800 MET.minutes/week; and a larger group (22%), whose initially moderate PA levels (around 800 MET.minutes/week) increased to almost 1800 MET.minutes/week by the age of 70. It is notable that median PA levels in every trajectory group (except the Low group – 0 MET.minutes/week) declined after age 70. Since lower PA increases the risk of adverse health outcomes [4], removing barriers to PA among mid-aged women with low or declining trajectories, and increasing opportunities for PA, may increase PA levels and consequently reduce chronic disease risk in older age [1, 3, 5].

These five trajectories contrast with the three relatively stable trajectories described for men using data from the British Regional Heart study [29]. The difference may reflect the fact that the British researchers conducted their first follow-up survey 12 years after baseline, and may have missed any earlier increases or decreases in PA, such as those we saw in about one-third of our sample during the first 12 years of follow-up in this ALSWH study.

When we examined the determinants of the different trajectories using the data from surveys 2 (age 47–52) and 5 (age 56–61), we found that BMI, and health were the strongest determinants of PA trajectories, especially in the Low-stable trajectory group where PA levels were remarkably low. Our findings clearly show markedly lower levels of overweight and obesity, and better PH and MH in the High-stable trajectory group. Moreover, women with increasing BMI were over-represented in three trajectory groups (Low-stable, Moderate-stable and High-declining) but not in the Moderate-increasing and High-stable groups. This is consistent with previous analyses of ALSWH data which illustrate the current weight gain epidemic in Australia, and indicates that women with high levels of PA are protected against high weight gain [47]. Worsening PH and MH were most marked in the Low- and Moderate-stable groups.

Many sociodemographic characteristics (including area of residence, education, ability to manage on income, hours of paid work, living with children, and caring duties) were also associated with the PA trajectories. Some of these associations were in line with expectations, while others were counter-intuitive. For example, as expected from our own and others’ research [15, 19], providing care (for grandchildren or others) was associated with declining PA. However, our findings on living with children (more likely in both the moderate-stable and increasing groups) and paid work (less likely in the declining group) point to the complex relationships between women’s paid work and caring roles, and PA patterns [48]. It is possible that women in paid work (with and without children) may have more opportunities and resources to support their leisure time activities.

Despite the fact that PA improves psychological well-being and sleep quality among menopausal women [49, 50], menopause symptoms may be a barrier to remaining active at this life stage. There were, however, few indications that menopause was associated with PA trajectory, except that the High-declining group were less likely to be post-menopausal.

### Strengths and limitations

The main strength of this study is that we used data from a nationally representative cohort with 21 years of follow-up. The ALSWH data, which were collected from 8 surveys at three-year intervals, provide a rare opportunity to examine long-term PA trajectories and the potential for non-linear changes over time. Another strength was that, in addition to the potential sociodemographic and health determinants of the trajectories, we assessed associations with changes in determinants over the first 12 years of follow-up.

The study has several limitations. First, participants with PA data from fewer than four surveys were excluded from the primary analysis, which may have introduced selection bias. However, we confirmed that the characteristics of the analytic and non-analytic samples were similar. One exception was for educational attainment, with over-representation of women with a University degree. This may explain the higher prevalence of meeting PA guidelines at age 47–52 in this cohort (54.6%), than in national survey estimates (44.1% for women age 45–54) [51]. However, the ALSWH women were age 47–52 in 1998, so our data may not be directly comparable with data from women of the same age today, due to modern day secular influences (period effects) [52]. We also acknowledge that the two groups may have differed in terms of unmeasured characteristics that may influence physical activity. Second, we obtained PA data using a self-reported questionnaire, which focuses mostly on leisure-time activity and recreational and transport-related walking. It does not include work-related activities [33]. Although self-report surveys may overestimate PA, our survey has acceptable reliability and validity [33, 53] and does not overestimate activity to the same extent as other self-report measures [53, 54]. Although use of accelerometers or Global Positioning System devices may reduce measurement bias, use of these devices to assess PA in 10,000 participants at three-year intervals for 21 years would be logistically and financially challenging. In any event, these measures were not widely available when the study began [55], and it is important to use the same PA measure over time in prospective studies [56]. Self-report may also introduce error in other variables, especially BMI. Although we have shown acceptable reliability and validity for the BMI measure in our cohort [37], we acknowledge that underweight women are more likely to over-report, and obese women tend to under-report their weight [37], and that there may be unreported changes in height at this life stage [57]. We accounted for missing values for height using protocols developed by the ALSWH researchers [58]. Third, we acknowledge that there may be complex bidirectional associations between BMI and health variables with PA, and that reverse causality cannot be ruled out [59]. However, our aim was to investigate predictors of PA trajectories, not causal relationships. Finally, although we used a wide range of sociodemographic, behavioural and health characteristics, additional potential determinants of PA trajectories may have been omitted.

## Conclusions

Trajectories of PA appear to remain stable, at low, moderate, or high levels, for the majority of mid-aged women. However, around 30% show marked changes in PA at this life stage (22% increasing and 8% declining). Our findings are encouraging, because most women maintained PA levels at or above current guidelines, and the ‘increasers’ seemed to be overcoming known constraints to PA, such as living with children, or caring for grandchildren. Of concern were the very low levels of PA in the Low-stable group, and the declining levels in the High-declining group, which, if maintained, are likely to increase the risk of adverse health outcomes [4, 60]. Increasing BMI and worsening health were notable in the low, moderate and declining PA trajectory groups. Our data suggest that promotion strategies, which address barriers to PA and increase capability, motivation, and opportunities [61] should be targeted to women with low or declining PA in mid-age, for improved health in older age [1, 3].

### Supplementary Information

**Additional file 1: Supplementary table 1. **Complete list of variables.** Supplementary figure 1. **Trajectories of meeting physical activity recommendation probability.** Supplementary table 2. **Goodness-of-fit and adequacy of the model (probability of meeting physical activity recommendation).** Supplementary table 3. **Goodness-of-fit and adequacy of the model (total physical activity).

**Additional file 2: **STROBE Statement—Checklist of items that should be included in reports of cohort studies.

## Data Availability

The dataset analysed in this study was provided by The Australian Longitudinal Study of Women’s Health. Information on how to access the data can be found at https://www.alswh.org.au/how-to-access-the-data/alswh-data.

## Abbreviations

AIC: Akaike information criterion
BIC: Bayesian information criterion
BMI: Body mass index
CI: Confidence interval
HRT: Hormone replacement therapy
IQR: Interquartile range
MH: Mental health
OCP: Oral contraceptive
OR: Odds ratio
PA: Physical activity
PH: Physical health
