# Supplementary material for: Trajectories of physical activity from mid to older age in women: 21 years of data from the Australian Longitudinal Study on Women’s Health
Source: Int J Behav Nutr Phys Act. 2024 Jan 8;21:4. doi: 10.1186/s12966-023-01540-z (PMC10773129; doi:10.1186/s12966-023-01540-z)
Supplement: Supplementary file 1 — Additional file 1: Supplementary table 1. Complete list of variables. Supplementary figure 1. Trajectories of meeting physical activity recommendation probability. Supplementary table 2. Goodness-of-fit and adequacy of the model (probability of meeting physical activity recommendation). Supplementary table 3. Goodness-of-fit and adequacy of the model (total physical activity). [file 12966_2023_1540_MOESM1_ESM.docx]

| **Supplementary table 1. Complete list of variables** | | |
| --- | --- | --- |
| **Variable name** | **Question or measure** | **Response options or categories** |
| **Country of birth** |  |  |
|  | In which country were you born? | Australia |
|  |  | United Kingdom |
|  |  | Italy |
|  |  | Greece |
|  |  | New Zealand |
|  |  | Vietnam |
|  |  | Other (Please specify) |
| **Area of residence** |  |  |
|  | Accessibility/Remoteness Index of Australia plus [1]  (derived from post code and geographic location of participant’s address). | Major city of Australia |
|  |  | Inner regional Australia |
|  |  | Outer regional Australia |
|  |  | Remote |
|  |  | Very remote Australia |
| **Educational attainment** | |  |
|  | What is the highest qualification you have completed? | School Certificate |
|  |  | Higher school certificate |
|  |  | Trade/Apprenticeship |
|  |  | Certificate/Diploma |
|  |  | University degree |
|  |  | Higher degree |
| **Marital status** |  |  |
|  | What is your present marital status? | Married |
|  |  | Defacto (opposite sex) |
|  |  | Defacto (same sex) |
|  |  | Separated |
|  |  | Divorced |
|  |  | Widowed |
|  |  | Single |
| **Ability to manage on income** | |  |
|  | How do you manage on the income you have available? | Easy |
|  |  | Not too bad |
|  |  | Difficult sometimes |
|  |  | Difficult always |
|  |  | Impossible |
| **Hours worked per week** | |  |
|  | How many hours do you normally spend in your paid work each week? | None |
|  |  | 1-15 h/wk |
|  |  | 16-24 h/wk |
|  |  | 25-34 h/wk |
|  |  | 35-40 h/wk |
|  |  | 41-48 h/wk |
|  |  | ≥49 h/wk |
| **Living with children age <18** | |  |
|  | How many people live with you now? | None |
|  | Children under 16 years  Children 16-18 years | One |
|  |  | Two |
|  |  | Three or more |
| **Provision of care for grandchildren** | |  |
|  | Do you regularly provide (unpaid) care for grandchildren? | Never |
|  |  | Occasionally |
|  |  | Weekly |
|  |  | Daily |
|  |  | Never |
| **Provision of care for someone requiring care** | |  |
|  | Do you regularly provide care or assistance (eg personal care, transport) to any other person because of their long-term illness, disability or frailty? | Occasionally |
|  |  | Weekly  Daily |
| **Menopause status** |  |  |
|  | Combination of hysterectomy, ovaries removed, hormone replacement therapy (HRT), oral contraceptive pill (OCP), menstrual bleeding, and irregular bleeding [2] | Hysterectomy only |
|  |  | Bilateral oophorectomy only |
|  |  | Hysterectomy and bilateral oophorectomy |
|  |  | HRT use |
|  |  | OCP use |
|  |  | Pre-menopausal |
|  |  | Peri-menopausal |
|  |  | Post-menopausal |
| **BMI** |  |  |
|  | Weight (kg) divided by the square of reported height (metres) [3]. BMI was classified based on WHO recommendations [4] | Underweight |
|  |  | Normal |
|  |  | Overweight |
|  |  | Obese |
| **Physical function** |  |  |
|  | Physical component summary score from the SF-36 [5] | Continuous variable; categorised into quartiles |
| **Mental health** |  |  |
|  | Mental component summary score from the SF-36 [5] | Continuous variable; categorised into quartiles |
| **Chronic conditions** |  |  |
|  | Have you ever been told by a doctor that you have?  Type 2 diabetes, heart disease, hypertension, stroke, low iron level, asthma, bronchitis/emphysema, osteoporosis, breast cancer, cervical cancer, skin cancer, depression, and anxiety | Never |
|  |  | Last 2 years |
|  |  | >2 years ago |
| **Smoking status** |  |  |
|  | Frequency and history of smoking [6] | Never-smoker |
|  |  | Ex-smoker |
|  |  | Smoker <10 cigarettes /day |
|  |  | Smoker 10-19 cigarettes /day |
|  |  | Smoker ≥20 cigarettes /day |
|  |  | Smoker unknown cigarettes/day |
| **Alcohol status** |  |  |
|  | Frequency and quantity of alcohol consumed [7] | Low risk drinker |
|  |  | Non-drinker |
|  |  | Rarely drinks |
|  |  | Risky drinker |
|  |  | High risk drinker |

Refenreces

1. Australian Longitudinal Study on Women’s Health. ARIA Scores: Index of accessibility/remoteness. Available from: <https://alswh.org.au/wp-content/uploads/2020/08/DDSSection5ARIA.pdf>. Accessed September 2023.

2. Australian Longitudinal Study on Women’s Health. Menopausal status Available from: <https://alswh.org.au/wp-content/uploads/2020/08/DDSSection2.2Menopause.pdf>. Accessed September 2023.

3. Australian Longitudinal Study on Women’s Health. Estimated Height – Young and Mid-aged. Available from: <https://alswh.org.au/wp-content/uploads/2020/08/DDSSection3Height.pdf>. Accessed September 2023.

4. Australian Longitudinal Study on Women’s Health. Body mass index – Young and Mid-aged. Available from: <https://alswh.org.au/wp-content/uploads/2020/08/DDSSection3BMI.pdf>. Accessed September 2023.

5. Mishra GD, Hockey R, Dobson AJ. A comparison of SF-36 summary measures of physical and mental health for women across the life course. Quality of Life Research. 2014;23(5):1515-21. doi:10.1007/s11136-013-0586-3.

6. Australian Longitudinal Study on Women’s Health. Smoking Status. Available from: <https://alswh.org.au/wp-content/uploads/2021/01/DDS_section2_5SmokingStatus.pdf>. Accessed September 2023.

7. Australian Longitudinal Study on Women’s Health. Alcohol Intake and Pattern of Alcohol Consumption. Available from: <https://alswh.org.au/wp-content/uploads/2020/08/DDSSection2.5AlcIntake.pdf>. Accessed September 2023.


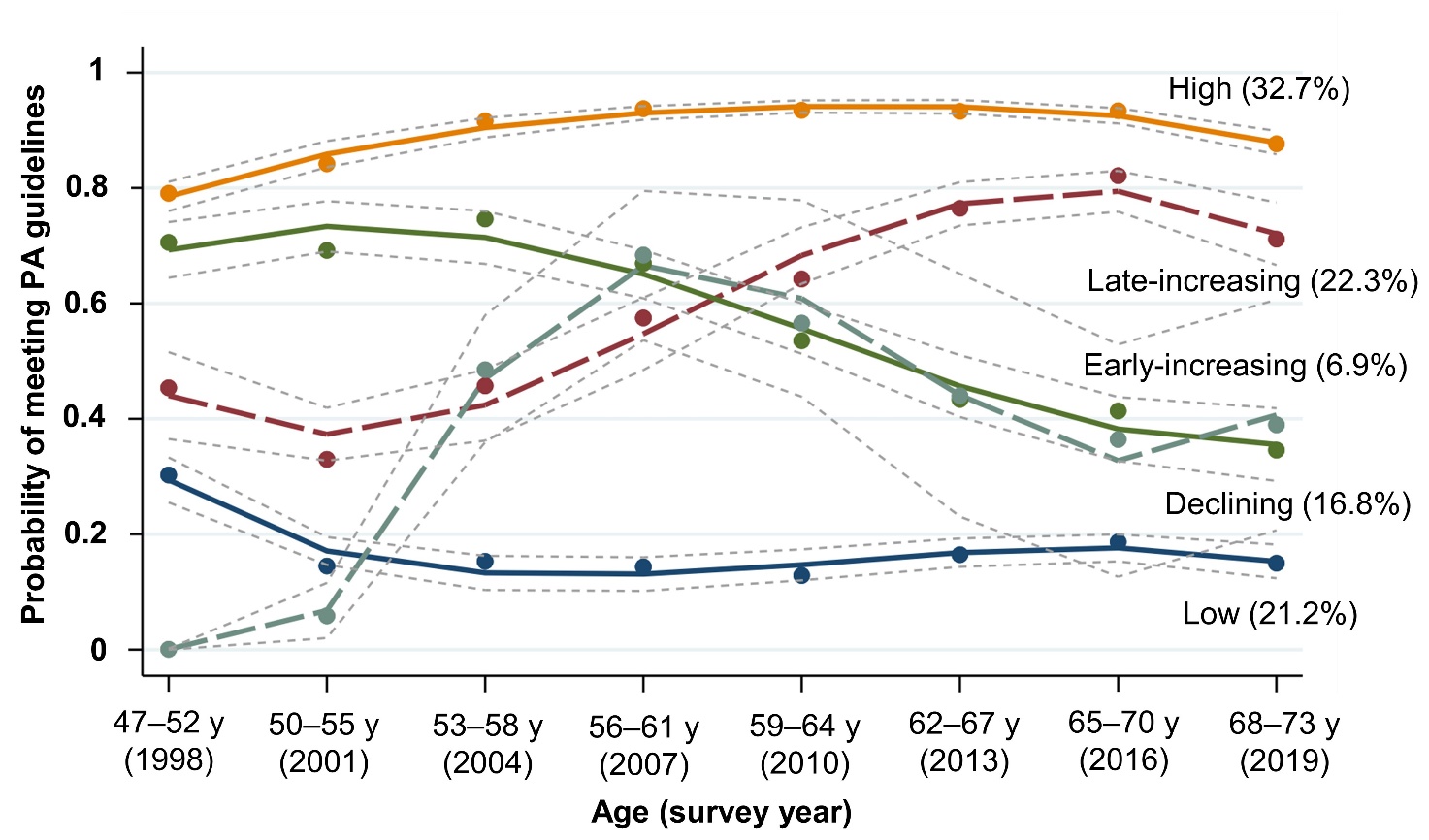


**Supplementary figure 1. Trajectories of probability of meeting physical activity recommendation**

Group-based trajectory model; ≥500 MET.minutes/week categorized as meeting physical activity recommendation.

PA, physical activity; y, year

| **Supplementary table 2.** **Goodness-of-fit and adequacy of the model (probability of meeting physical activity recommendation)** | | | | | | | | |
| --- | --- | --- | --- | --- | --- | --- | --- | --- |
| **Group** | **Polynomial** | **BIC** | **AIC** | **Log-likelihood** | **Trajectories** | **Estimated probability of group membership** | **Average posterior probabilities** | **Odds of correct classification** |
| 2 | Linear | -43457.55 | -43439.43 | -43434.43 | - | - | - | - |
| 2 | Quadratic | -43312.33 | -43286.96 | -43279.96 | - | - | - | - |
| 2 | Cubic | -43278.65 | -43246.04 | -43237.04 | - | - | - | - |
| 3 | Linear | -43156.03 | -43127.04 | -43119.04 | - | - | - | - |
| 3 | Quadratic | -42988.19 | -42948.33 | -42937.33 | - | - | - | - |
| 3 | Cubic | -42962.36 | -42911.64 | -42897.64 | - | - | - | - |
| 4 | Linear | -42957.8 | -42917.94 | -42906.94 | - | - | - | - |
| 4 | Quadratic | -42860.37 | -42806.02 | -42791.02 | - | - | - | - |
| 4 | Cubic | -42817.45 | -42748.61 | -42729.61 | - | - | - | - |
| 5 | Linear | -42933.24 | -42882.51 | -42868.51 | - | - | - | - |
| 5 | Quadratic | -42818.85 | -42750.01 | -42731.01 | - | - | - | - |
| **5** | **Cubic** | **-42781.04** | **-42694.08** | **-42670.08** | Low | 21.2 | 0.768 | 12.33 |
|  |  |  |  |  | Declining | 16.8 | 0.643 | 8.92 |
|  |  |  |  |  | Early-increasing | 6.9 | 0.508 | 13.85 |
|  |  |  |  |  | Late-increasing | 22.3 | 0.603 | 5.29 |
|  |  |  |  |  | High | 32.7 | 0.802 | 8.3 |
| BIC, Bayesian information criterion; AIC, Akaike information criterion | | | | | | | | |
| Five trajectories of the cubic polynomials model were selected. | | | | | | | | |

| **Supplementary table 3. Goodness-of-fit and adequacy of the model (total physical activity)** | | | | | | |  |  |
| --- | --- | --- | --- | --- | --- | --- | --- | --- |
| **Group** | **Polynomial** | **BIC** | **AIC** | **Log-likelihood** | **Trajectories** | **Estimated probability of group membership** | **Average posterior probabilities** | **Odds of correct classification** |
| 2 | Linear | -509324.9 | -509303.1 | -509297.1 | - | - | - | - |
| 2 | Quadratic | -509297.1 | -509112.8 | -509104.8 | - | - | - | - |
| 2 | Cubic | -509112.2 | -509076.0 | -509066.0 | - | - | - | - |
| 3 | Linear | -509066.0 | -507758.9 | -507749.9 | - | - | - | - |
| 3 | Quadratic | -507583.0 | -507539.6 | -507527.6 | - | - | - | - |
| 3 | Cubic | -507549.4 | -507495.1 | -507480.1 | - | - | - | - |
| 4 | Linear | -507480.1 | -507309.0 | -507297.0 | - | - | - | - |
| 4 | Quadratic | -507129.2 | -507071.2 | -507055.2 | - | - | - | - |
| 4 | Cubic | -507095.5 | -507023.0 | -507003.0 | - | - | - | - |
| 5 | Linear | -506941.0 | -506886.6 | -506871.6 | - | - | - | - |
| 5 | Quadratic | -506687.3 | -506614.8 | -506594.8 | - | - | - | - |
| **5** | **Cubic** | **-506650.0** | **-506559.4** | **-506534.4** | Low-stable | 13.3 | 0.825 | 30.51 |
|  |  |  |  |  | Moderate-stable | 50.4 | 0.844 | 5.32 |
|  |  |  |  |  | Moderate-increasing | 22.0 | 0.785 | 12.96 |
|  |  |  |  |  | High-declining | 7.7 | 0.741 | 34.25 |
|  |  |  |  |  | High-stable | 6.6 | 0.865 | 90.25 |
| BIC, Bayesian information criterion; AIC, Akaike information criterion | | | | | | | | |
| Five trajectories of the cubic polynomials model were selected. | | | | | | | | |
